# A Lifecourse Approach to Long-Term Sickness Absence—A Cohort Study

**DOI:** 10.1371/journal.pone.0036645

**Published:** 2012-05-03

**Authors:** Max Henderson, Charlotte Clark, Stephen Stansfeld, Matthew Hotopf

**Affiliations:** 1 Department of Psychological Medicine, King's College London Institute of Psychiatry, London, United Kingdom; 2 Centre for Psychiatry, Wolfson Institute for Preventive Medicine, Barts and the London School of Medicine and Dentistry, Queen Mary University of London, London, United Kingdom; The University of Hong Kong, Hong Kong

## Abstract

**Background:**

Most research on long-term sickness absence has focussed on exposure to occupational psychosocial risk factors such as low decision latitude. These provide an incomplete explanation as they do not account for other relevant factors. Such occupational risk factors may be confounded by social or temperamental risk factors earlier in life.

**Methods:**

We analysed data from the 1958 British Birth Cohort. Long-term sickness absence was defined as receipt of Incapacity Benefit/Severe Disablement Allowance at age 42. In those in employment aged 33 we examined the effects of psychological distress, musculoskeletal symptoms, and low decision latitude. These were then adjusted for IQ, educational attainment, and the presence of early life somatic and neurotic symptoms.

**Results:**

Low decision latitude predicted subsequent long-term absence, and this association remained, albeit reduced, following adjustment for psychological distress and musculoskeletal symptoms at age 33. Low decision latitude was no longer associated with long-term absence when IQ and educational attainment were included. Adjusting for early life somatic and neurotic symptoms had little impact.

**Discussion:**

A greater understanding of the ways in which occupational risk factors interact with individual vulnerabilities across the life-course is required. Self reported low decision latitude might reflect the impact of education and cognitive ability on how threat, and the ability to manage threat, is perceived, rather than being an independent risk factor for long-term sick leave. This has implications for policy aimed at reducing long-term sick leave.

## Introduction

Long term sickness absence is costly for individuals and the economy. In the UK 180 million working days were lost due to sickness in 2009 [1] costing the economy £17 billion [1] There are also substantial costs to individuals in terms of loss of income, dignity and reduced social participation. Reducing the number of people claiming work-related benefits is an important target for policy-makers [2]–[4]. Despite diverse benefit systems, many developed countries have large proportions of the workforce on sick leave costing considerable sums in disability benefits [5]. Much of the literature in the field comes from Scandinavia [6] – Norway, for example has 11.4% of its working age population claiming disability benefits [7]

Most sickness absence, especially long term absence, is attributable to symptom-based conditions - mainly musculoskeletal and common mental disorders [8]–[11]. There is little association between the severity of a disorder and the risk it will lead to absence from work [12], [13]. Whilst non-workplace factors such as the nature of home life and the need to provide childcare have been associated with sickness absence [14], most research has focussed on occupational risk factors.

Physical workload only weakly predicts sickness absence [15]–[19] even for musculoskeletal disorders such as low back pain. Adverse psychosocial work environments, as measured by Job Strain [20] or Effort Reward Imbalance [21] for example, have been associated with psychiatric disorders [22], [23] and sickness absence [24]–[27]. However not all studies show this [28], [29]. Policy is often focussed on improving such environments [30]. Most studies rely on self-reported measures of the psychosocial workplace environment. Few studies have used objective measures and those that have report conflicting results. Virtanen used ward-overcrowding as a proxy for high demands in nurses' psychosocial work environment and showed this was independently associated with increased consumption of antidepressant medication [31]. However Stansfeld's study using Whitehall II data showed that when more objective measures of job demands are used the association between the work environment and psychological morbidity was attenuated [32]. Iennaco failed to find an association between externally rated measures of job strain and depression [33]. Further, an objective change in workplace conditions – downsizing – has been reported to both increase [34] and decrease [35] sickness absence, although these responses may be subject to type of employment contract and local social conditions. Rehkopf [36] in a further analysis of Whitehall II data suggested that whilst sickness absence was differentially predicted by subjective and objective measures of job demands, no such difference was observed for decision latitude.

We suggest that personality and coping strategies such as fear-avoidance [37], catastrophising responses [38] and individual expectations [12], [18], [39] may be responsible for both the perception of stress and subsequent sickness absence [40]. These results are difficult to interpret, however, as these coping strategies may have developed following previous adverse experiences at work, blurring the distinction between ‘individual’ and ‘workplace’ risk factors. A life-course approach may avoid such difficulties as in birth cohorts “upstream” risk factors can be assessed prior to an individual starting work.

In this paper we aimed to determine the extent to which the impact of self-reported low decision latitude on long term sickness absence could be explained by both complaints of psychological distress and musculoskeletal symptoms and by individual risk factors assessed earlier in life.

## Methods

### Sample

The data were from the National Child Development Study, which includes 98% of all births in the UK in one week in March 1958. It has been described in detail elsewhere [41]. Information was obtained from parents and participants at ages 7, 11 and 16. Participant interviews were carried out at ages 23, 33 and 42.

### Outcome

There is no standard definition of long term sickness absence in the UK. Data were available on receipt of work-related benefits at age 42. Participants were identified as long term absentees if they were in receipt of Incapacity Benefit (IB) and Severe Disablement Allowance (SDA) which are long term benefits designed to replace lost income [42] for those unable to work due to ill health. IB is the most common of these and is awarded to those who are no longer entitled to Statutory Sick Pay, most often, though not always, because they have been off work due to sickness or disability for greater than 28 weeks. SDA, scrapped in 2001, was awarded to those who were unable to work for 28 weeks and were ineligible for IB because for example they had made insufficient National Insurance contributions. For new claimants, IB has recently been replaced by the Employment and Support Allowance (ESA).

### Explanatory variables

#### Decision latitude

Four questions were asked about the psychosocial work environment - (1) ‘my work requires me to keep learning new things’ (2) ‘my work is monotonous because I always do the same things’ (3) ‘I can only take breaks at certain times’ and (4) ‘I am able to vary the pace at which I work’. Participants responded on a 5-point likert scale from ‘true’ to ‘not true’. Participant responses were reversed where necessary to reflect negative characteristics and dichotomised (0/1). The responses were then summed (range 0–4) such that higher scores represent lower (worse) decision latitude. This measure of the psychosocial work environment has been previously described by Matthews [43], [44] although it was referred to as a measure of the broader concept of Job Strain. As only statement 1 contains an element of demands as opposed to control/decision latitude we felt this was better described as a measure of decision latitude.

#### Adult covariables

Established risk factors for long term sickness absence were identified at age 33. Psychological distress was assessed with the Malaise Inventory [45], the validity of which is well established [46]. Scores over 7 (“Malaise case”) suggest high levels of distress [47]. Participants who answered positively to questions on the inventory about either ‘arthritis or rheumatism’ or ‘back pain lasting more than 1 day’ in the last year were included as having symptoms of a musculoskeletal disorder. The 4-item CAGE questionnaire was completed and those scoring at least 2 were classed as ‘problem drinkers’ [48]. Occupational social class and highest educational attainment were recorded at age 33.

#### Early life covariables

Socio-demographic data were taken from various time-points. Social class and gender were recorded at birth (from information on the occupation of the mother's husband). The results of the General Ability test [49], a measure of IQ, at age 11, were divided into quartiles. Aspects of childhood temperament have been shown to be associated with long term sickness absence in adult life [50]. Parental responses (‘no’/‘sometimes’/‘frequently’) to statements (1) ‘is child miserable or tearful’ (2) ‘does child worry about many things’ at age 11 were included. Parents were also asked about somatic health complaints by the child. Responses to questions about whether their child complains of headaches or abdominal pain (at age 11) were dichotomised into ‘yes’ or ‘no’.

### Statistical analyses

Data were analysed using STATA, version 9.2 (Stata, College Station, TX, USA). In order to avoid the possibility that results might be influenced by the inclusion of individuals too ill or disabled ever to be employed, analyses were initially restricted to those who reported they were in work, education or caring for a family aged 33 (93%). Univariable associations with the outcome ‘Long term sickness absence’ were calculated using logistic regression. The numbers (%) long term sick in each category are shown together with the odds ratios (95% confidence intervals). Individual odds ratios for each category within a variable were calculated using logistic regression analysis, using indicator variables. P values are for the z test of the null hypothesis that the logistic coefficient equals zero when no indicator variable was used. Where there are more than two categories in a variable this can be interpreted as a test for trend. All variables, regardless of the level of univariable association, were entered into a multivariable logistic regression analysis. Variables were entered into the analysis as groups in a sequence decided *a priori*. Model 1 comprises low decision latitude adjusted for sex. Model 2 includes the established risk factors for long term sickness absence. In model 3, childhood IQ and adult educational attainment were added. Finally the remaining early life variables were added in model 4.

### Sample attrition

Like many longitudinal studies the NCDS suffers from loss to follow up. Of a possible 18558 participants, 1090 had died and 1321 emigrated by 2000, leaving a potential sample of 16147. Of which 11419 (71%) were included. Non-participation during adult life is associated with lower socioeconomic status, lower IQ, lower educational attainment and scoring as a case on the Malaise Inventory.

To address the problem of missing data multiple imputation using the ICE programme in STATA (Version 9.2) was performed. This is a principled method of imputation that inflates neither the sample size nor the power of the study [51]. All variables reported here were included in the imputation equations. All participants except those who had died or emigrated during the course of the study were included in the imputation. Ten cycles of the imputation were run and analyses indicated that the measures were stable across the imputations [51]. Parameter estimates from the ten imputations were estimated using the MICOMBINE function in STATA [52].

### Ethics approval

This is secondary analysis of existing data

## Results

Multiple imputation produced a dataset of 16147, of whom 15053 (93%) were identified as being in work, education or caring for a family aged 33. Of these 431 (2.9% 95% CI 2.2%–3.6%) were categorised as long term sick.

Table 1 shows the univariable associations with being long term sick aged 42. Strong associations are shown with the established risk factors as well as with standard socio-demographic variables such as social class in childhood and adulthood.

**Table 1:** Univariable association of exposure variables with long term sickness absence in 2000.

Variable		N (%)	n (%) on long term benefits	OR (95% CI)	p-value
**Sex**	Male	7483 (49.7%)	190 (2.5%)	0.8 (0.6–1.0)	p = 0.08
	Female	7570 (50.3%)	241 (3.2%)		
**Social class at birth**	I	664 (4.4%)	(0.7%)	1	p<0.001 (trend)
	II	1948 (12.9%)	33 (1.7%)	2.5 (0.7–9.1)	
	III	8845 (58.8%)	274 (3.1%)	4.8 (1.3–17.2)	
	IV	1815 (12.1%)	55 (3.0%)	4.7 (1.2–17.8)	
	V	1781 (11.8%)	65 (3.6%)	5.6 (1.6–20.3)	
**Social class aged 33**	I	557 (3.7%)	9 (1.7%)	1	p = 0.03 (trend)
	II	2118 (14.1%)	46 (2.2%)	1.4 (0.5–3.4)	
	III	7777 (51.7%)	220 (2.8%)	1.8 (0.7–4.5)	
	IV	3781 (25.1%)	125 (3.3%)	2.1 (0.8–5.4)	
	V	820 (5.4%)	31 (3.8%)	2.4 (0.7–7.7)	
**Cognitive ability**	1st quartile (most able)	3604 (23.9%)	54 (1.5%)	1	p<0.001 (trend)
	2nd quartile	3900 (25.9%)	77 (2.0%)	1.3 (0.8–2.1)	
	3rd quartile	3782 (25.2%)	125 (3.3%)	2.2 (1.5–3.3)	
	4th quartile (least able)	3767 (25.0%)	175 (4.6%)	3.2 (2.1–4.8)	
**Highest educational attainment age 33**	Degree or higher	1821 (12.1%)	21 (1.2%)	1	p<0.001 (trend)
	A level	4204 (27.9%)	86 (2.1%)	1.8 (1.0–4.4)	
	Level	5195 (34.5%)	143 (2.8%)	2.4 (1.3–4.5)	
	CSE Grade 2–5	1958 (13.0%)	79 (4.0%)	3.6 (2.1–6.3)	
	No Qualifications	1875 (12.5%)	103 (5.5%)	5.0 (2.8–9.0)	
**Problem drinker (CAGE > = 2)**	Yes	557 (3.7%)	27 (4.9%)	1.8 (0.9–3.4)	p = 0.1
	No	14496 (96.3%)	404 (2.8%)		
**Miserable or tearful age11**	No	8945 (59.4%)	236 (2.6%)	1	p = 0.08 (trend)
	Sometimes	5560 (36.9%)	173 (3.1%)	1.2 (0.9–1.5)	
	Frequently	548 (3.7%)	22 (4.0%)	1.5 (0.9–2.7)	
**Worries about many things age11**	No	7054 (46.9%)	190 (2.7%)	1	p = 0.06 (trend)
	Sometimes	5960 (39.6%)	162 (2.7%)	1 (0.8–1.3)	
	Frequently	2039 (13.5%)	78 (3.8%)	1.4 (1.1–2.0)	
**Recurrent headaches age11**	Yes	2359 (15.7%)	87 (3.7%)	1.4 (1.0–2.0)	p = 0.08
	No	12694 (84.3%)	344 (2.7%)		
**Recurrent abdominal pain age11**	Yes	1642 (10.9%)	66 (4.0%)	1.5 (1.1–2.1)	p = 0.02
	No	13411 (89.1%)	365 (2.7%)		
**Depression age 33 (“Malaise case”)**	Yes	948 (6.3%)	73 (7.7%)	3.2 (2.3–4.5)	p<0.001
	No	14105 (93.7%)	358 (2.5%)		
**Musculoskeletal symptoms age33**	Yes	7820 (51.9%)	275 (3.5%)	1.7 (1.3–2.2)	p<0.001
	No	7233 (48.1%)	156 (2.2%)		
**Decision Latitude score age 33**	0 (highest)	6394 (42.5%)	146 (2.3%)	1	p = 0.001 (trend)
	1	5583 (37.1%)	165 (3.0%)	1.3 (1.0–1.7)	
	2	2410 (16.0%)	93 (3.9%)	1.7 (1.2–2.4)	
	3	562 (3.7%)	24 (4.2%)	1.9 (1.1–3.3)	
	4 (lowest)	104 (0.7%)	4 (4.0%)	1.7 (0.5–6.1)	

The multivariable analyses are shown in Table 2. In analyses adjusted only for sex reporting low decision latitude was a strong predictor of subsequent long term sickness absence, although in contrast to other ordered categorical variables, such as social class and IQ, there was no clear dose-response relationship with long term sickness absence. Whilst an overall effect of low decision latitude remained after adjustment for psychological distress and musculoskeletal symptoms, the effect size was attenuated and there did not appear to be a greater effect with higher scores (model 2). The effect sizes were more substantially attenuated by including childhood IQ and educational attainment in the model (model 3). Including symptom reports from early life had relatively little further effect (model 4).

**Table 2:** Multivariable analysis: risk factors for long term sickness absence in 2000.

Variable		Model 1		Model 2		Model 3		Model 4	
		OR (95% CI)	P value	OR (95% CI)	P value	OR (95% CI)	P value	OR (95% CI)	P value
Decision latitude aged 33 (total score)	0	1	p = 0.003	1	p = 0.04	1	p = 0.35	1	p = 0.37
	1	1.3 (0.8,1.9)		1.2 (0.8,1.8)		1.1 (0.7,1.7)		1.1 (0.7,1.7)	
	2	1.7 (1.2,2.5)		1.5 (1.1,2.2)		1.3 (0.9,1.9)		1.3 (0.9,1.9)	
	3	1.9 (1.2,3.2)		1.6 (1.0,2.7)		1.3 (0.8,2.2)		1.3 (0.8,2.2)	
	4	1.4 (0.6,3.5)		1.2 (0.5,3.0)		0.9 (0.3,2.2)		0.9 (0.4,2.2)	
Sex	Male	0.8 (0.6,1.0)	P = 0.04	0.8 (0.6,1.0)	P = 0.04	0.8 (0.6,1.0)	P = 0.05	0.8 (0.6,1.0)	P = 0.07
Malaise case age 33				2.8 (2.0,4.0)	p<0.001	2.3 (1.7,3.3)	p<0.001	2.2 (1.6,3.2)	p<0.001
Musculo-skeletal symptoms age 33				1.4 (1.0,1.8)	P = 0.04	1.3 (1.0,1.8)	P = 0.05	1.3 (1.0,1.8)	P = 0.05
Social Class age 33	I			1	P = 0.09	1	P = 0.97	1	P = 0.98
	II			1.2 (0.5,2.9)		1.0 (0.4,2.5)		1.0 (0.4,2.5)	
	III			1.4 (0.6,3.4)		1.0 (0.4,2.4)		0.9 (0.4,2.3)	
	IV			1.6 (0.6,3.9)		0.9 (0.4,2.4)		0.9 (0.4,2.3)	
	V			1.8 (0.7,5.1)		1.1 (0.4,3.0)		1.0 (0.4,2.9)	
CAGE case age 33				1.7 (0.9,3.2)	P = 0.12	1.7 (0.9,3.3)	P = 0.09	1.8 (0.9,3.4)	P = 0.08
Educational attainment	Degree					1	P = 0.003	1	P = 0.004
	A level					1.4 (0.7,2.8)		1.3 (0.6,2.6)	
	O level					1.7 (0.8,3.5)		1.5 (0.7,3.2)	
	CSE 2–5					2.0 (1.0,4.2)		1.8 (0.9,3.8)	
	None					2.5 (1.2,5.2)		2.2 (1.0,4.8)	
Cognitive ability (1^st^ quartile = most able)	1st quartile					1	p = 0.002	1	p = 0.002
	2nd quartile					1.2 (0.7,1.9)		1.1 (0.7,1.8)	
	3rd quartile					1.6 (0.9,2.7)		1.5 (0.9,2.6)	
	4th quartile					2.0 (1.2,3.2)		1.9 (1.1,3.1)	
Social class at birth	I							1	p = 0.55
	II							2.0 (0.6,6.6)	
	III							2.7 (0.9,8.2)	
	IV							2.3 (0.7,7.6)	
	V							2.5 (0.8,8.0)	
“Worries” age 11	No							1	P = 0.16
	Sometimes							1.1 (0.9,1.5)	
	Frequently							1.3 (0.9,2.0)	
“Miserable” age 11	No							1	P = 0.98
	Sometimes							1.0 (0.8,1.3)	
	Frequently							0.9 (0.5,1.8)	
Headaches age 11								1.2 (0.8,1.6)	P = 0.45
Tummyaches age 11								1.2 (0.8,1.8)	P = 0.32

All the analyses were also carried out on the original, non-imputed data set (data not shown). These showed a slightly weaker association of long term absence with psychological distress and a stronger association with the highest category of job but otherwise yielded broadly similar results.

## Discussion

Although in unadjusted analyses low decision latitude was strongly associated with later long term sickness absence, the effect was attenuated when symptoms of musculoskeletal disorder and psychological distress were included, and disappeared when educational attainment and childhood IQ were included. Our results suggest that the association between self-reported low decision latitude and subsequent long term sick leave may result from such “upstream” individual risk factors.

A number of possible limitations need to be considered. Although the measure of decision latitude has been used elsewhere, it is unvalidated and crude compared to what would be available from a full Karasek Inventory [53]. Moreover in the 1958 cohort this measure was available at a single time point – a more accurate assessment of the impact of an adverse psychosocial work environment would have been obtained were repeated measures available. It is possible that the experience of low decision latitude might precipitate complaints of psychological distress or musculoskeletal disorder but we are unable to address this using these data.

When attempting to understand the role of possible confounding and mediating factors we were only able to use variables that had been collected, and as such there may be residual confounding due either to incomplete adjustment using the variables provided, or because information we would have liked to have used was not available – more detailed parental occupational information for example. The data collections were spaced out in time. Whilst this may lessen “questionnaire fatigue” in participants it means that we have no information about what has happened to them in the intervening periods. For example although we have identified individuals on long term benefits at age 42, we do not know how long they have been on them or what happened to the participants between 33 and 42.

We used multiple imputation to counter the significant problems caused by loss to follow-up. It is unlikely that this method provides complete adjustment for non-participation [54]. As with any cohort study it is possible our findings apply only to this particular time or to this particular cohort.Symptoms of musculoskeletal disorder and psychological distress were assessed at the same time as decision latitude. As such we are not able to separate out confounding from mediation in their relationship with long term sickness absence. Both are possible. Having psychological distress may be a confounding, or alternative, explanation as it could make it more likely that an individual perceives the external world as threatening, and will reduce the individual's perception that they are able to change this. As such they might report lower levels of decision latitude, but the “actual” reason for long term sick leave was the underlying psychiatric disorder. Alternatively low decision latitude, when combined with high demands constitutes ‘high job strain’ which has been repeatedly to be associated with subsequent psychiatric disorder. As such low decision latitude may have contributed to the development of a psychiatric disorder which was the reason for the long term sick leave. In this case the psychiatric disorder would have mediated the relationship between low decision latitude and long term sick leave.

Although there is a growing literature on the relationship between the psychosocial work environment and psychiatric disorder [27], [55] we have not attempted to separate long term sickness absence into that attributed to physical or psychological problems. This is deliberate and based on the clinical observation that many factors contribute to an individual taking sick leave [40]. It has long been recognised that the sick leave of psychologically distressed individuals attracts many different labels [56], [57]. That such distress is evident in long term absentees 9 years earlier suggests taking sick leave is often a process not an event.

Although highest educational attainment was assessed at 33 we think it is unlikely that it might mediate the relationship between low decision latitude and long term sick leave mainly because although assessed at age 33 by and large it reflects educational achievement much earlier in life. This can be stated even more categorically for IQ measured in childhood. We suggest that the effect of adjusting for educational attainment and IQ is best understood as confounding. It is likely that those with lower IQ and lower educational attainment end up in jobs which are characterised by lower decision latitude. Such individuals may be less able to adapt successfully when they become ill as their qualifications and skills may be more limited. There is also a role for individual perceptions being a risk factor for long term sick leave has been suggested before. One possibility is that lower cognitive ability and lower educational attainment contribute to an individual perceiving his environment differently, and also perceiving his ability to change his environment differently. A similar model has been proposed to explain the association between lower IQ and mortality. We have recently demonstrated, using data from 3 British birth cohorts, that cognitive ability measured in childhood is an independent predictor of long term sick leave [58].

Very few studies have considered the role of childhood risk factors for adult occupational outcomes. Both Upmark et al [59] and Harkonmäki et al [60] used childhood data recalled in later life rather than contemporaneously collected data. Gravseth et al [61] examined routinely collected data for associations between aspects of childhood adversity and early ill health retirement, showing that low educational attainment was a strong risk factor. This is however the first study to examine a range of potential early life risk factors and established adult risk factors for long term sickness absence.

Much of the current discussion about sickness absence, and certainly that about “work stress” appears based on a simple “one-hit” model whereby healthy individuals come to work, are made ill somehow, and go off sick. This limited view ignores the fact that most individuals exposed to the work environments in question do not go off sick - indeed work has many health benefits [62]. Moreover it ignores the many factors which contribute to the complex decision to take time off. A more sophisticated model is needed where the importance of workplace and non-workplace factors is recognised, but so are individual vulnerabilities [63]

The UK population has never been healthier in terms of survival, and workplaces have never been safer, yet record numbers are claiming disability benefits – the paradox of health [64]. Occupational health research started with a toxicological approach where symptoms at work were due to workplace exposures which had to be identified then eliminated or avoided. Heightened awareness of the apparent risk was beneficial. Whilst this has been very successful for many occupational diseases, most disorders which affect occupational function today are multi-factorial symptom-based conditions. Rarely do they arise as a direct effect of workplace exposure. Increased awareness leads to more complaints [65]. These disorders reflect a combination of external triggers and individual vulnerabilities occurring in cultural circumstances where satisfaction with personal health is low despite high expectations of medical care [66]. Our findings suggest policies to reduce long term sickness absence which focus mainly on individual reports of the psychosocial work environment may produce disappointing results Intervention strategies must pay heed to individual vulnerabilities if they are to be successful [67].
